# 3-Substituted *N*-Benzylpyrazine-2-carboxamide Derivatives: Synthesis, Antimycobacterial and Antibacterial Evaluation

**DOI:** 10.3390/molecules22030495

**Published:** 2017-03-21

**Authors:** Lucia Semelková, Ondřej Janďourek, Klára Konečná, Pavla Paterová, Lucie Navrátilová, František Trejtnar, Vladimír Kubíček, Jiří Kuneš, Martin Doležal, Jan Zitko

**Affiliations:** 1 Faculty of Pharmacy in Hradec Králové, Charles University, Heyrovského 1203, 500 05 Hradec Králové, Czech Republic; JANDO6AA@faf.cuni.cz (O.J.); konecna@faf.cuni.cz (K.K.); navratl2@faf.cuni.cz (L.N.); trejtnarf@faf.cuni.cz (F.T.); kubicek@faf.cuni.cz (V.K.); kunes@faf.cuni.cz (J.K.); dolezalm@faf.cuni.cz (M.D.); 2 Department of Clinical Microbiology, University Hospital, Sokolská 581, 500 05 Hradec Králové, Czech Republic; pavla.paterova@fnhk.cz

**Keywords:** pyrazinamide derivatives, benzylamines, antibacterial activity, antimycobacterial activity, cytotoxicity, enoyl-ACP-reductase, molecular docking

## Abstract

A series of substituted *N*-benzyl-3-chloropyrazine-2-carboxamides were prepared as positional isomers of 5-chloro and 6-chloro derivatives, prepared previously. During the aminolysis of the acyl chloride, the simultaneous substitution of chlorine with benzylamino moiety gave rise to *N*-benzyl-3-(benzylamino)pyrazine-2-carboxamides as side products, in some cases. Although not initially planned, the reaction conditions were modified to populate this double substituted series. The final compounds were tested against four mycobacterial strains. *N*-(2-methylbenzyl)-3-((2-methylbenzyl)amino)pyrazine-2-carboxamide (**1a**) and *N*-(3,4-dichlorobenzyl)-3-((3,4-dichlorobenzyl)amino)pyrazine-2-carboxamide (**9a**) proved to be the most effective against *Mycobacterium tuberculosis* H37Rv, with MIC = 12.5 μg·mL^−1^. Compounds were screened for antibacterial activity. The most active compound was 3-chloro-*N*-(2-chlorobenzyl)pyrazine-2-carboxamide (**5**) against *Staphylococcus aureus* with MIC = 7.81 μM, and *Staphylococcus epidermidis* with MIC = 15.62 μM. HepG2 in vitro cytotoxicity was evaluated for the most active compounds; however, no significant toxicity was detected. Compound **9a** was docked to several conformations of the enoyl-ACP-reductase of *Mycobacterium tuberculosis*. In some cases, it was capable of *H*-bond interactions, typical for most of the known inhibitors.

## 1. Introduction

Every living organism is bound to evolve in order to survive. Although it is a natural process spanning millions of years, in the case of pathogenic microorganisms, this adaptation for survival is rather undesirable from a human point of view. Microorganisms have developed a range of protective mechanisms to deactivate, remove, or otherwise circumvent the toxicity of anti-infective compounds [1]. The appearance of resistance is positively linked to the frequency of use of such an anti-infective. Practically, resistance to any clinically used anti-infective drug will develop sooner or later [2]. Increasing rates of microbial resistance to both commonly used and reserved agents highlight the necessity to develop new effective compounds.

According to the WHO Global Tuberculosis Report 2016, it was estimated that there were 10.4 million new tuberculosis (TB) cases and 1.4 million TB associated deaths in 2015. Although TB incidence rates have been slowly declining for several consecutive years and an estimated 43 million lives were saved between 2000 and 2014 through effective diagnosis and treatment, the presence of resistant forms and co-infection with HIV pose a considerable threat to successful treatment. The percentage of new TB cases that were multidrug-resistant (MDR-TB) was 3.3% in 2014, whereas HIV-positive patients represented 12% of the 9.6 million people who developed TB [3]. Pyrazinamide (PZA), as a fist-line antitubercular drug, has gained an irreplaceable role in TB therapy and has been subjected to various chemical modifications [4,5,6,7]. Although PZA has been used for over sixty years [8], it is still not completely known how PZA acts in a mycobacterial cell [8,9,10,11,12].

**Figure 1:**
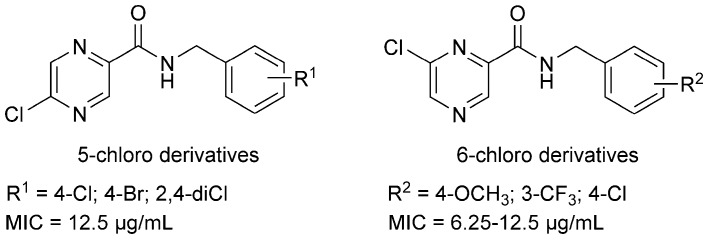
Structures of previously reported chloropyrazine-2-carboxamide derivatives with in vitro antimycobacterial activity (*M. tuberculosis* H3Rv).

5-Chloropyrazinamide, as a fatty acid synthase I (FAS I, involved in mycolic acids biosynthesis) inhibitor [10], was a pattern for a series of 5- or 6-chloropyrazine-2-carboxamides with *N*-benzylamino substitution (Figure 1), prepared and previously reported by Servusová et al. [13,14]. 5-Cl derivatives with 4-Cl, 4-Br, or 2,4-diCl substitution on the phenyl ring exerted antimycobacterial activity with a minimum inhibitory concentration (MIC) of 12.5 μg·mL^−1^, and 6-Cl derivatives with 4-OCH_3_, 3-CF_3_, or 4-Cl substitution on the phenyl ring showed an MIC of 6.25–12.5 μg·mL^−1^ [13,14]. As reported in this article, compounds **1**–**13** comprising 3-Cl-pyrazine moiety were originally designed as positional isomers with respect to the chloro substitution on the pyrazine ring, in order to study the effect of positional isomerism on anti-infective activity.

However, dibenzyl substituted derivatives **1a**–**13a**, which initially originated as side-products of intended monosubstituted derivatives, turned out to be more effective compounds.

As a complementary test, all of our compounds were subject to initial screening for in vitro activity against clinically important Gram-positive and Gram-negative bacteria, including methicillin-resistant *Staphylococcus aureus* (MRSA). Any potential hit compound against MRSA would be highly appreciated as MRSA has become one of the most feared pathogens in the last few decades [14].

## 2. Results and Discussion

### 2.1. Chemistry

**Scheme 1:**
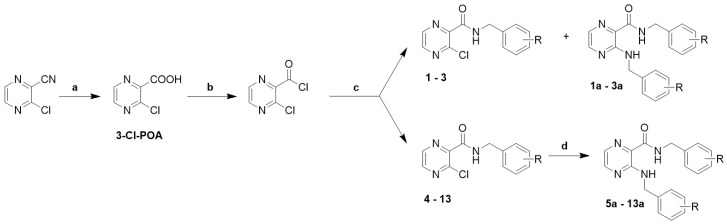
Synthesis of compounds **1**–**13** and **1a**–**13a**. *Reagents and conditions*: (**a**) 10% aq. sol. NaOH, reflux 7 h, then 10% aq. sol. HCl; (**b**) SOCl_2_, DMF, toluene, 95 °C, 1 h; (**c**) substituted benzylamine, TEA, acetone, RT, overnight; (**d**) substituted benzylamine, pyridine, methanol, MW: 150 °C, 30 min, 100 W.

Substituted *N*-benzyl-3-chloropyrazine-2-carboxamides **1**–**13** were prepared by a three-step procedure (Scheme 1), according to Ref. [15]. The initial 3-chloropyrazine-2-carbonitrile (Fluorochem Ltd., Derbysshire, UK) was hydrolysed in 10% (*m*/*m*) aqueous NaOH solution. After 7 h, the reaction mixture was acidified with 10% HCl to pH 3, to obtain crystals of 3-chloropyrazine-2-carboxylic acid (**3-Cl-POA**), which were then filtered and dried. In the following step, 3-Cl-POA was treated with SOCl_2_ in the presence of a catalytic amount of *N*,*N-*dimethylformamide (DMF) in toluene under reflux for one hour, producing 3-chloropyrazine-2-carbonyl chloride, which was reacted with substituted benzylamine in the presence of triethylamine (TEA) in acetone at room temperature overnight, to obtain **1**–**13**. In the process of amide bond formation with 2-methyl-, 4-methyl-, and 4-methoxybenzylamine, corresponding *N-*benzyl-3-(benzylamino)pyrazine-2-carboxamides **1a**–**3a** were formed simultaneously with **1**–**3** as desired side-products in a molar ratio of approximately 2:3, with an excess of **1a**–**3a**. The side-products could be easily detected by TLC and easily separated by flash-chromatography. We decided to complete the disubstituted series. *N*-Benzyl-3-(benzylamino)pyrazine-2-carboxamides **5a**–**13a** were afforded via the aminodehalogenation of **5**–**13**, with the corresponding substituted benzylamine and pyridine in methanol under microwave conditions (150 °C, 30 min, power output 100 W, focused field) (Scheme 1d). However, compounds **4a** (R^1,2^ = 2,4-OCH_3_), **8a** (R^1,2^ = 2,4-Cl) and **12a** (R^1,2^ = 3-CF_3_) could not be prepared under the chosen conditions. This might have been due to the electron-withdrawing effects of CF_3_ substitution and/or the steric hindrance of large substituents in the *ortho* position of the phenyl ring. All prepared compounds (Table 1) were characterized by ^1^H-, ^13^C-NMR, and IR spectroscopy, their melting point, and elemental analysis. All results were fully in accordance with the proposed structures.

### 2.2. Lipophilicity

**Figure 2:**
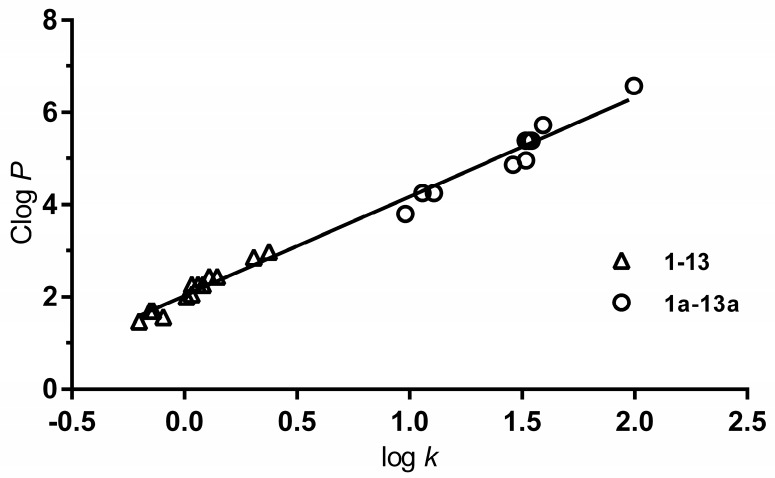
Plot of calculated Clog*P* on experimentally measured log *k* values.

**Figure 3:**
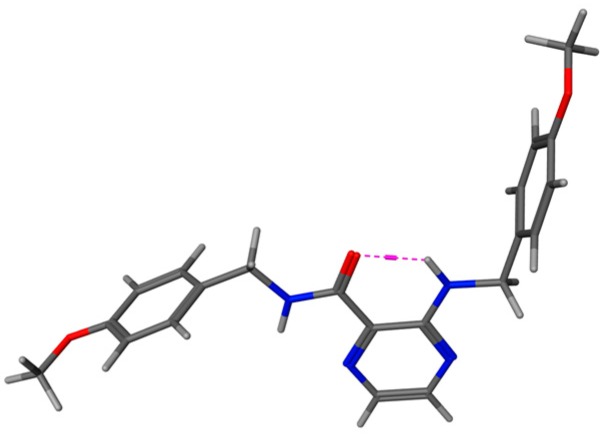
Visualization of the intramolecular hydrogen bond (compound **3a**).

The lipophilicity parameters log *P* and Clog*P* were calculated for compounds by ChemDraw Ultra, ver. 14.0. Capacity factor *k*, expressed as log *k*, was experimentally measured using RP-HPLC. The values of the lipophilicity parameters Clog*P* and log *k* are presented in Table 1. The relationship between the calculated Clog*P* and measured log *k* is linear, with a regression equation:

(1) Clog*P* = (2.149 ± 0.053) log *k* + (2.29 ± 0.052); R^2^ = 0.987; s = 0.185; F = 1622; n = 23

see Figure 2. Clog*P* takes into consideration the intramolecular hydrogen bond formed between carbonyl oxygen and amine hydrogen (Figure 3). Although the precise algorithm of Clog*P* calculation is not publically known, its consideration of intramolecular *H*-bond formation can be documented by shifting the benzylamino substituent to various positions of the pyrazine core and comparing the obtained Clog*P* values. For example, in the case of compound **3a** (3-benzylamino derivative) and its 5- and 6-positional isomers, the calculated Clog*P* values are 3.802, 3.725, and 3.752. The increased value for the vicinal disubstituted derivative is caused by the intramolecular *H-*bonding, which decreases the probability to form compound-water *H-*bonds, and therefore, lowers its hydrophilicity. On the contrary, the intramolecular interactions are not recognized by log *P* and all the three positional isomers appeared to share the same predicted lipophilicity of log *P* = 2.35. As a result, the calculated Clog*P* values of the presented compounds have a stronger correlation with the measured log *k* than the log *P* values.

### 2.3. Biological Evaluation

#### 2.3.1. In Vitro Antimycobacterial Activity

All prepared compounds were tested against *Mycobacterium tuberculosis* H37Rv (*M. tbc*), as well as non-tuberculous strains of *M. avium* and *M. kansasii*. Additionally, *M. smegmatis* was used as a fast growing model organism with a highly lipophilic cell wall, similar in composition to the one of *M. tbc.* In vitro screening was performed by a Microplate Alamar Blue Assay (MABA) on whole cells. Results were expressed as the minimum inhibitory concentration (MIC) in μg·mL^−1^. The results for *M. tbc* and *M. smegmatis* are shown in Table 1.

From the series of 3-chloro derivatives (**1**–**13**), the only substance exhibiting significant activity against *M. tbc* H37Rv was compound **3**, with a 4-OCH_3_ substitution on the benzene ring with MIC = 25 μg·mL^−1^ (90 μM). Relative to the 5-Cl and 6-Cl derivatives published before [13,14] (Figure 1), the reposition of chlorine to position 3 led to a decrease or loss of antimycobacterial activity against *M. tbc* H37Rv. The most effective compounds from the 3-benzylamino-*N*-benzylpyrazine-2-carboxamide series (**1a**–**13a**) were structures **1a** (R^1,2^ = 2-CH_3_) and **9a** (R^1,2^ = 3,4-diCl) with MIC = 12.5 μg·mL^−1^ (**1a** 36 μM; **9a** 27 μM) against *M. tbc*. The introduction of the second phenyl ring (**1a**, **2a**, **9a**) often led to an increase of antimycobacterial activity when compared to the corresponding single phenyl derivatives (**1**, **2**, **9**). The presence of electron-donating substituents (2-CH_3_, 4-CH_3_, 4-OCH_3_) proved advantageous for the biological effect. Electron-withdrawing substituents (F, Cl, CF_3_) did not produce active compounds, even in double phenyl compounds, except for **9a** (2,4-diCl).

The presented compounds were inactive against *M. kansasii* and *M. avium*, even at the highest tested concentration of 100 μg·mL^−1^ (or 50 μg·mL^−1^ for **7a**, due to its lower solubility in DMSO).

Five compounds, namely **3**, **6**, **8**, **10**, and **12**, showed modest activity against fast growing *M. smegmatis* with MIC = 125 μg·mL^−1^. All these compounds belong to the first series of *N*-benzyl-3-chloropyrazine-2-carboxamides. On the contrary to the activity against *M. tbc*, the activity against *M. smegmatis* was mutilated by the insertion of the second aromatic ring.

#### 2.3.2. Antibacterial Activity

Antibacterial assays were performed against eight clinically significant strains (see Experimental Section). Interesting antibacterial activity against gram-positive *Staphylococcus aureus*, methicillin-resistant *S. aureus* (MRSA), and *S. epidermidis*, was found in the series of 3-chloro derivatives with a single benzene core (see Table 2). Compound **5** (R^1^ = 2-Cl) exerted the highest activity against *S. aureus* with MIC = 7.81 μM, and against *S. epidermidis* with MIC = 15.62 μM. Previously published 5-Cl and 6-Cl positional isomers did not show any antibacterial activity in tested concentrations up to 500 μM [13,14].

The insertion of the second aromatic ring generally decreased the antibacterial activity (with the exception of compound **3a**). From the series containing two aromatic moieties (**1a**–**13a**), only compounds with 4-OCH_3_ (**3a**) and 4-CF_3_ (**13a**) displayed antibacterial activity. The most effective compound against MRSA was **3a** (R^1,2^ = 4-OCH_3_), with MIC = 62.5 μM.

Compounds not shown in Table 2 did not produce any significant activity against tested bacterial strains.

#### 2.3.3. Antifungal Activity

Additionally, the compounds were tested against eight fungal strains (see Experimental Section). No compounds from the presented series showed any activity in antifungal assays up to the highest tested concentration (500 μM).

#### 2.3.4. Cytotoxicity

The in vitro cytotoxicity of the most active compounds **5**, **1a**, **3a**, and **9a**, was evaluated using the standard human hepatocellular carcinoma cell line HepG2. The results of the experiments are presented as a concentration, which reduces the viability of the cell population to 50% of the maximal viability, IC_50_ (Table 3). The limited solubility of the compounds **1a**, **3a**, and **9a** in the cell culture medium, did not allow a valid calculation of the IC_50_ value. Compound **5** (R^1^ = 2-Cl), the most active compound against methicillin sensitive *S. aureus*, exerted IC_50_ = 847.2 μM, leading to a superior selectivity index, SI = IC_50_/MIC = 108.

### 2.4. Docking Studies

Based on molecular docking studies, various *N*-benzylpyrazine-2-carboxamides have been previously suggested as potential inhibitors of mycobacterial enoyl-ACP-reductase (InhA) [4], an essential enzyme in the synthesis of mycolic acids and the primary target of the first-line antitubercular isoniazid. In such PZA derivatives, the carbonyl oxygen of the carboxamide moiety was predicted to form an *H*-bond network to Tyr158 and 2′-OH of the ribose of the NAD^+^ cofactor, which is a typical interaction pattern for most of the direct InhA inhibitors derived from triclosan [16].

Most of the compounds presented in this paper with in vitro activity against *M. tuberculosis* were disubstituted derivatives with two large benzyl substituents on two adjacent positions of the pyrazine core. We were interested to find out whether such sterically demanding derivatives would be able to fit in the active site of InhA in a manner similar to smaller PZA derivatives with a single aryl substituent. Therefore, we performed molecular docking of the most active dibenzyl derivative **9a** into various conformations of InhA, differing in the size of the active site cavity, which is formed by the highly flexible substrate-binding loop (Figure S1, Supplementary Materials). Not surprisingly, **9a** was not able to fit into closed conformations of InhA (pdb: 2X23; 3FNF) and did not show the expected ligand-receptor interactions. On the other hand, when an opened conformation of the InhA receptor was used (pdb: 4R9S, 4TZK, or 5G0S), we were able to identify two different binding modes for **9a**, with scores similar to the score of the co-crystalized ligands and, more importantly, with ligand-receptor interactions known to be typical for InhA inhibitors. For a detailed depiction of the predicted binding modes of **9a** to InhA and experimental details on the computational procedures, see the Supplementary Materials.

Although the results of molecular docking are not enough to confirm the inhibition of InhA as the mechanism of the action of derivative **9a**, we have shown that even such sterically demanding derivatives are able to mimic the poses and interactions of known InhA inhibitors. The selection of a suitable structure of InhA with an open conformation was crucial in this docking study.

## 3. Experimental Section

### 3.1. General

The starting 3-chloropyrazine-2-carbonitrile was purchased from Fluorochem Ltd. (Hadfield, Derbysshire, UK). Other chemicals were of a reagent or higher grade of purity and were purchased from Sigma-Aldrich (Steinheim, Germany). The progress of the reactions was monitored by Thin Layer Chromatography (TLC) (TLC Silica gel 60 F_254_, Merck, Darmstadt, Germany) with UV detection using wavelength of 254 nm. Microwave-assisted reactions were performed in a CEM Discover microwave reactor with a focused field (CEM Corporation, Matthews, NC, USA) connected to an Explorer 24 autosampler (CEM Corporation), and the equipment was run under CEM’s Synergy™ software (version 1.38, CEM Corporation) for setting and monitoring the conditions of the reactions. The temperature of the reaction mixture was monitored indirectly, by an internal infrared sensor. All obtained products were purified by a preparative flash chromatograph CombiFlash^®^ Rf (Teledyne Isco Inc., Lincoln, NE, USA). The type of elution was gradient, using a mixture of hexane (LachNer, Neratovice, Czech Republic) and ethyl acetate (Penta, Prague, Czech Republic) as the mobile phase. Silica gel (0.040–0.063 nm, Merck, Darmstadt, Germany) was used as the stationary phase. NMR spectra were taken with spectrometers Varian Mercury-VxBB 300, with frequencies 299.95 MHz for ^1^H and 75.43 MHz for ^13^C or Varian VNMR S500 (499.87 MHz for ^1^H and 125.71 MHz for ^13^C) (Varian Corporation, Palo Alto, CA, USA). Chemical shifts were reported in ppm (δ) and were referred indirectly to tetramethylsilane via the solvent signal. Infrared spectra were recorded with a FT-IR Nicolet 6700 spectrometer (Thermo Scientific, Waltham, MA, USA), using attenuated total reflectance (ATR) on Ge crystal. Elemental analyses were performed using a Micro Cube Elemental Analyzer (Elementar Analysensysteme GmbH, Hanau, Germany). Melting points were assessed by SMP3 Stuart Scientific (Bibby Sterling Ltd., Staffordshire, UK) in an open capillary and are uncorrected. The lipophilicity parameters log *P* and Clog*P* were calculated by software CS ChemBioDraw Ultra 14.0 (CambridgeSoft, Cambridge, MA, USA). The yields of the products refer to chromatographically pure products after all purification steps and are given in percentage to theoretical yield related to the starting acid.

### 3.2. Chemistry

#### 3.2.1. 3-Chloropyrazine-2-carboxylic Acid (3-Cl-POA)

The starting compound 3-chloropyrazine-2-carbonitrile (35.8 mmol) was dispersed in a 10% solution of sodium hydroxide (125 mmol, 3.5 equiv.) in a round bottom flask. The reaction mixture was stirred and heated under reflux in an oil bath for 7 h. The progress of the reaction was monitored by TLC in the system butanol/acetic acid/water (4:1:5). The reaction mixture was acidified by 10% solution of hydrochloric acid to pH 3. Resulting crystals of 3-chloropyrazine-2-carboxylic acid were suctioned. The melting point and ^1^H-NMR corresponded with the literature [17].

#### 3.2.2. General Synthetic Procedure for *N*-Benzyl-3-chloropyrazine-2-carboxamides **1**–**13**

A total of 0.3 g of 3-Cl-POA (1.9 mmol) was dispersed in dry toluene with thionyl chloride (0.4 mL, 5.7 mmol, 3 equiv.) and 1–2 drops of *N*,*N-*dimethylformamide (DMF) as a catalyst. The reaction mixture was stirred and heated in a round bottom flask in an oil bath under a condenser, at 95 °C for 1 h. The solvent was evaporated in vacuo and the residue was azeotroped with dry toluene (3 × 20 mL). The acyl chloride was used for the following step, without purification. 

The whole amount of the 3-chloropyrazine-2-carbonyl chloride prepared in the previous step was dissolved in dry acetone. An appropriate benzylamine (5.7 mmol, 3 equiv., with respect to the starting acid), along with triethylamine (1.9 mmol, 1 equiv.), were added to the reaction mixture and stirred at laboratory temperature overnight. The progress of the reaction was checked by TLC in system hexane/ethyl acetate (1:1 or 2:1). The reaction mixture was adsorbed to silica by removing the solvents in vacuo and the product was purified by flash chromatography using gradient elution with ethyl acetate in hexane. For compounds **1**–**3**, corresponding *N*-benzyl-3-(benzylamino)pyrazine-2-carboxamides **1a**–**3a** were formed simultaneously in a molar ratio of approximately 2:3, with an excess of **1a**–**3a**. R_f_ in hexane/ethyl acetate 1:1 mobile phase: **1a**—0.85, **1**—0.49, **2a**—0.84, **2**—0.51, **3a**—0.71, and **3**—0.40. After flash-chromatography, compounds **2**, **2a**, **3**, **3a**, **4**, **8**, and **13** were recrystallized from EtOH/H_2_O.

#### 3.2.3. General Synthetic Procedure for *N*-Benzyl-3-(benzylamino)pyrazine-2-carboxamides (**5a**–**7a**, **9a**–**11a** and **13a**)

Compounds **5a**–**7a**, **9a**–**11a**, and **13a** were synthetized using a microwave reactor with a focused field. Corresponding *N-*benzyl-3-chloropyrazine-2-carboxamide (0.6 mmol) was dissolved in methanol (3 mL) and appropriate benzylamine (1.8 mmol, 3 equiv.), along with pyridine (40 mg, 0.6 mmol, 1 equiv.) as a base, were added. Our previous observations revealed that triethylamine (TEA) as a base cannot be used for microwave reactions. During the procedure, TEA is partially decomposed (probably to diethylamine and similar species, which may act as undesired nucleophiles in the dehalogenation reaction). The use of pyridine as the base combined with benzylamines was experimentally verified in previous projects [18]. Conditions for synthesis were 150 °C, 30 min, and 100 W. The progress of the reactions was monitored by TLC in system hexane/ethyl acetate 1:1. The reaction mixture was adsorbed on silica by removing the solvents in vacuo and the product was purified by flash chromatography using gradient elution with ethyl acetate (0–100%) in hexane. Products **7a** and **10a** were recrystallized from EtOH/H_2_O.

### 3.3. Analytical Data of Compounds

*3-Chloro-N-(2-methylbenzyl)pyrazine-2-carboxamide* (**1**). Ochre solid. Yield 18%; m.p. 94.5–96.5 °C; IR (ATR-Ge, cm^−1^): 3292, 1652 (C=O, CONH); ^1^H-NMR (500 MHz, CDCl_3_) δ 8.52 (d, *J* = 2.3 Hz, 1H, pyr.), 8.44 (d, *J* = 2.3 Hz, 1H, pyr.), 7.73 (bs, 1H, NH), 7.34–7.17 (m, 4H, Ar), 4.65 (d, *J* = 5.7 Hz, 2H, CH_2_), 2.39 (s, 3H, CH_3_); ^13^C-NMR (126 MHz, CDCl_3_) δ 161.67, 148.25, 145.62, 143.10, 140.51, 136.48, 135.17, 130.54, 128.65, 127.91, 126.22, 41.89, 19.04; Elemental analysis: calc. for C_13_H_12_ClN_3_O (MW 261.71): 59.66% C, 4.62% H, 16.06% N; found 59.69% C, 4.91% H, 15.85% N.

*N-(2-Methylbenzyl)-3-((2-methylbenzyl)amino)pyrazine-2-carboxamide* (**1a**). Yellow-brown solid. Yield 26%; m.p. 89.6–91.4 °C; IR (ATR-Ge, cm^−1^): 3395, 3321, 1659 (C=O, CONH); ^1^H-NMR (500 MHz, CDCl_3_) δ 8.96 (bs, 1H, NH), 8.21 (d, *J* = 2.4 Hz, 1H, pyr.), 8.13 (t, *J* = 5.8 Hz, 1H, NH), 7.67 (d, *J* = 2.4 Hz, 1H, pyr.), 7.39–7.16 (m, 8H, Ar), 4.71 (d, *J* = 5.5 Hz, 2H, CH_2_), 4.60 (d, *J* = 5.8 Hz, 2H, CH_2_), 2.40 (d, *J* = 17.6 Hz, 6H, CH_3_; ^13^C-NMR (126 MHz, CDCl_3_) δ 166.21, 154.53, 146.49, 136.47, 136.32, 136.22, 135.54, 130.49, 130.32, 129.52, 128.26, 128.04, 127.73, 127.28, 126.42, 126.20, 126.02, 42.51, 41.30, 19.11, 19.03; Elemental analysis: calc. for C_21_H_22_N_4_O (MW 346.43): 72.81% C, 6.40% H, 16.17% N; found 72.44% C, 6.49% H, 16.35% N.

*3-Chloro-N-(4-methylbenzyl)pyrazine-2-carboxamide* (**2**). Pale brown solid. Yield 17%; m.p. 126.2–129.1 °C; IR (ATR-Ge, cm^−1^): 3303, 1655 (C=O, CONH); ^1^H-NMR (500 MHz, CDCl_3_) δ 8.52 (d, *J* = 2.3 Hz, 1H, pyr.), 8.45 (d, *J* = 2.3 Hz, 1H, pyr.), 7.86 (bs, 1H, NH), 7.30–7.24 (m, 2H, Ar), 7.21–7.13 (m, 2H, Ar), 4.61 (d, *J* = 5.9 Hz, 2H, CH_2_, 2.35 (s, 3H, CH_3_); ^13^C-NMR (126 MHz, CDCl_3_) δ 161.77, 148.37, 145.63, 143.12, 140.49, 137.43, 134.53, 129.42, 127.94, 43.54, 21.07; Elemental analysis: calc. for C_13_H_12_ClN_3_O (MW 261.71): 59.66% C, 4.62% H, 16.06% N; found 59.47% C, 5.07% H, 15.69% N.

*N-(4-Methylbenzyl)-3-((4-methylbenzyl)amino)pyrazine-2-carboxamide* (**2a**). Pale yellow solid. Yield 25%; m.p. 145.8–147.6 °C; IR (ATR-Ge, cm^−1^): 3373, 3344, 1650 (C=O, CONH); ^1^H-NMR (300 MHz, CDCl_3_) δ 9.03 (t, *J* = 5.7 Hz, 1H, NH), 8.22 (s, 1H, NH), 8.18 (d, *J* = 2.4 Hz, 1H, pyr.), 7.64 (d, *J* = 2.4 Hz, 1H, pyr.), 7.30–7.21 (m, 4H, Ar), 7.18–7.12 (m, 4H, Ar), 4.67 (d, *J* = 5.7 Hz, 2H, CH_2_), 4.55 (d, *J* = 6.0 Hz, 2H, CH_2_), 2.34 (d, *J* = 2.9 Hz, 6H, CH_3_); ^13^C-NMR (75 MHz, CDCl_3_) δ 166.28, 154.52, 146.45, 137.17, 136.66, 135.80, 134.93, 129.52, 129.35, 129.19, 127.68, 127.49, 126.46, 44.10, 42.89, 21.07; Elemental analysis: calc. for C_21_H_22_N_4_O (MW 346.43): 72.81% C, 6.40% H, 16.17% N; found 72.47% C, 6.40% H, 15.73% N.

*3-Chloro-N-(4-methoxybenzyl)pyrazine-2-carboxamide* (**3**). Brown solid. Yield 11%; m.p. 107.7–109.3 °C; IR (ATR-Ge, cm^−1^): 3276, 1648 (C=O, CONH); ^1^H-NMR (300 MHz, CDCl_3_) δ 8.51 (d, *J* = 2.3 Hz, 1H, pyr.), 8.43 (d, *J* = 2.3 Hz, 1H, pyr.), 7.82 (bs, 1H, NH), 7.32–7.24 (m, 2H, Ar), 6.91–6.84 (m, 2H, Ar), 4.58 (d, *J* = 5.8 Hz, 2H, CH_2_), 3.79 (s, 3H, CH_3_); ^13^C-NMR (75 MHz, CDCl_3_) δ 161.76, 159.16, 148.37, 145.65, 143.14, 140.51, 129.65, 129.34, 114.15, 55.28, 43.26.; Elemental analysis: calc. for C_13_H_12_ClN_3_O_2_ (MW 277.71): 56.23% C, 4.36% H, 15.13% N; found 56.64% C, 4.84% H, 14.82% N.

*N-(4-Methoxybenzyl)-3-((4-methoxybenzyl)amino)pyrazine-2-carboxamide* (**3a**). Yellow-brown solid. Yield 15%; m.p. 83.1–85.7 °C; IR (ATR-Ge, cm^−1^): 3392, 3335, 1652 (C=O, CONH); ^1^H-NMR (300 MHz, CDCl_3_) δ 8.99 (bs, 1H, NH), 8.19 (bs, 1H, NH), 8.17 (d, *J* = 2.4 Hz, 1H, pyr.), 7.64 (d, *J* = 2.4 Hz, 1H, pyr.), 7.34–7.22 (m, 4H, Ar), 6.91–6.82 (m, 4H, Ar), 4.63 (d, *J* = 5.6 Hz, 2H, CH_2_), 4.51 (d, *J* = 5.9 Hz, 2H, CH_2_), 3.79 (s, 6H, CH_3_); ^13^C-NMR (75 MHz, CDCl_3_) δ 166.28, 159.03, 158.76, 154.52, 146.46, 131.00, 130.09, 129.55, 129.06, 129.06, 128.87, 126.51, 114.11, 113.97, 55.28, 43.86, 42.63; Elemental analysis: calc. for C_21_H_22_N_4_O_3_ (MW 378.43): 66.65% C, 5.86% H, 14.81% N; found 66.95% C, 6.12% H, 14.35% N.

*3-Chloro-N-(2,4-dimethoxybenzyl)pyrazine-2-carboxamide* (**4**). Pale brown solid. Yield 14%; m.p. 115.2–117.3 °C; IR (ATR-Ge, cm^−1^): 3275, 1640 (C=O, CONH); ^1^H-NMR (300 MHz, DMSO) δ 8.97 (t, *J* = 5.7 Hz, 1H, NH), 8.68 (d, *J* = 2.5 Hz, 1H, pyr.), 8.61 (d, *J* = 2.5 Hz, 1H, pyr.), 7.19 (d, *J* = 8.3 Hz, 1H, Ar), 6.58–6.47 (m, 2H, Ar), 4.37 (d, *J* = 5.8 Hz, 2H, CH_2_), 3.79 (s, 3H, CH_3_), 3.74 (s, 3H, CH_3_); ^13^C-NMR (75 MHz, DMSO) δ 163.74, 160.09, 157.95, 148.54, 145.32, 145.16, 142.61, 129.04, 118.24, 104.54, 98.46, 55.63, 55.40, 37.43.; Elemental analysis: calc. for C_14_H_14_ClN_3_O_3_ (MW 307.73): 54.64% C, 4.59% H, 13.65% N; found 54.95% C, 4.96% H, 13.66% N.

*3-Chloro-N-(2-chlorobenzyl)pyrazine-2-carboxamide* (**5**). Pale brown solid. Yield 46%; m.p. 101.7–102.8 °C; IR (ATR-Ge, cm^−1^): 3362, 1672 (C=O, CONH); ^1^H-NMR (500 MHz, CDCl_3_) δ 8.53 (d, *J* = 2.3 Hz, 1H, pyr.), 8.48 (d, *J* = 2.3 Hz, 1H, pyr.), 8.10 (bs, 1H, NH), 7.51–7.46 (m, 1H, Ar), 7.41–7.37 (m, 1H, Ar), 7.28–7.23 (m, 2H, Ar), 4.74 (d, *J* = 6.3 Hz, 2H, CH_2_); ^13^C-NMR (126 MHz, CDCl_3_) δ 161.84, 148.37, 145.74, 142.83, 140.55, 135.03, 133.73, 130.39, 129.54, 129.13, 127.14, 41.67; Elemental analysis: calc. for C_12_H_9_Cl_2_N_3_O (MW 282.12): 51.09% C, 3.22% H, 14.89% N; found 50.85% C, 3.58% H, 14.53% N.

*3-Chloro-N-(3-chlorobenzyl)pyrazine-2-carboxamide* (**6**). Brown solid. Yield 56%; m.p. 92.5–95.0 °C; IR (ATR-Ge, cm^−1^): 3279, 1660 (C=O, CONH); ^1^H-NMR (500 MHz, CDCl_3_) δ 8.54 (d, *J* = 2.3 Hz, 1H, pyr.), 8.47 (d, *J* = 2.3 Hz, 1H, pyr.), 8.01 (bs, 1H, NH), 7.34 (d, *J* = 2.0 Hz, 1H, Ar), 7.31–7.22 (m, 3H, Ar), 4.62 (d, *J* = 6.1 Hz, 2H, CH_2_); ^13^C-NMR (126 MHz, CDCl_3_) δ 161.92, 148.40, 145.83, 142.74, 140.54, 139.66, 134.53, 130.00, 127.82, 127.80, 125.94, 43.07; Elemental analysis: calc. for C_12_H_9_Cl_2_N_3_O (MW 282.12): 51.09% C, 3.22% H, 14.89% N; found 51.42% C, 3.69% H, 14.61% N.

*3-Chloro-N-(4-chlorobenzyl)pyrazine-2-carboxamide* (**7**). Brown solid. Yield 63%; m.p. 133.5–135.2 °C; IR (ATR-Ge, cm^−1^): 3382, 1669 (C=O, CONH); ^1^H-NMR (300 MHz, CDCl_3_) δ 8.53 (d, *J* = 2.4 Hz, 1H, pyr.), 8.45 (d, *J* = 2.3 Hz, 1H, pyr.), 7.94 (bs, 1H, NH), 7.34–7.24 (m, 4H, Ar), 4.61 (d, *J* = 6.1 Hz, 2H, CH_2_); ^13^C-NMR (75 MHz, CDCl_3_) δ 161.90, 148.42, 145.81, 142.80, 140.52, 136.15, 133.48, 129.22, 128.87, 43.01; Elemental analysis: calc. for C_12_H_9_Cl_2_N_3_O (MW 282.12): 51.09% C, 3.22% H, 14.89% N; found 50.81% C, 3.50% H, 14.74% N.

*3-Chloro-N-(2,4-dichlorobenzyl)pyrazine-2-carboxamide* (**8**). Ochre solid. Yield 58%; m.p. 99.5–100.4 °C; IR (ATR-Ge, cm^−1^): 3295, 1655 (C=O, CONH); ^1^H-NMR (300 MHz, DMSO) δ 9.38 (t, *J* = 6.0 Hz, 1H, NH), 8.73 (d, *J* = 2.5 Hz, 1H, pyr.), 8.66 (d, *J* = 2.4 Hz, 1H, pyr.), 7.63 (t, *J* = 1.2 Hz, 1H, Ar), 7.46 (d, *J* = 1.3 Hz, 2H, Ar), 4.53 (d, *J* = 5.9 Hz, 2H, CH_2_); ^13^C-NMR (75 MHz, DMSO) δ 163.93, 147.60, 145.72, 145.33, 142.63, 134.94, 133.25, 132.69, 130.38, 128.85, 127.59, 40.53; Elemental analysis: calc. for C_12_H_8_Cl_3_N_3_O (MW 316.57): 45.53% C, 2.55% H, 13.27% N; found 45.72% C, 2.79% H, 12.96% N.

*3-Chloro-N-(3,4-dichlorobenzyl)pyrazine-2-carboxamide* (**9**). Brown solid. Yield 49%; m.p. 121.5–124.5 °C; IR (ATR-Ge, cm^−1^): 3241, 1648 (C=O, CONH); ^1^H-NMR (300 MHz, CDCl_3_) δ 8.55 (d, *J* = 2.3 Hz, 1H, pyr.), 8.47 (d, *J* = 2.3 Hz, 1H, pyr.), 8.02 (bs, 1H, NH), 7.47–7.36 (m, 2H, Ar), 7.28–7.16 (m, 1H, Ar), 4.59 (d, *J* = 6.1 Hz, 2H, CH_2_); ^13^C-NMR (75 MHz, CDCl_3_) δ 161.96, 148.48, 145.97, 142.51, 140.54, 137.96, 132.73, 131.69, 130.67, 129.67, 127.16, 42.54; Elemental analysis: calc. for C_12_H_8_Cl_3_N_3_O (MW 316.57): 45.53% C, 2.55% H, 13.27% N; found 45.41% C, 2.77% H, 12.87% N.

*3-Chloro-N-(2-fluorobenzyl)pyrazine-2-carboxamide* (**10**). Pale brown solid. Yield 46%; m.p. 116.7–117.9 °C; IR (ATR-Ge, cm^−1^): 3320, 1661 (C=O, CONH); ^1^H-NMR (500 MHz, CDCl_3_) δ 8.53 (d, *J* = 2.2 Hz, 1H, pyr.), 8.47 (d, *J* = 2.2 Hz, 1H, pyr.), 8.01 (bs, 1H, NH), 7.44 (dt, *J* = 7.6 Hz *J* = 2.0 Hz, 1H, Ar), 7.32–7.26 (m, 1H, Ar), 7.15–7.10 (m, 1H, Ar), 7.10–7.05 (m, 1H, Ar), 4.70 (d, *J* = 5.9 Hz, 2H, CH_2_); ^13^C-NMR (125 MHz, CDCl_3_) δ 161.9, 161.1 (d, *J* = 247.1 Hz), 148.4, 145.8, 142.9, 140. 5, 130.4 (d, *J* = 3.9 Hz), 129.5 (d, *J* = 7.7 Hz), 124.6 (d, *J* = 15.3 Hz), 124.4 (d, *J* = 3.9 Hz), 115.4 (d, *J* = 21.0 Hz), 37.7 (d, *J* = 3.9 Hz); Elemental analysis: calc. for C_12_H_9_ClFN_3_O (MW 265.67): 54.25% C, 3.41% H, 15.82% N; found 54.61% C, 3.89% H, 15.59% N.

*3-Chloro-N-(4-fluorobenzyl)pyrazine-2-carboxamide* (**11**). Brown solid. Yield 55%; m.p. 115.0–117.9 °C; IR (ATR-Ge, cm^−1^): 3403, 1661 (C=O, CONH); ^1^H-NMR (300 MHz, CDCl_3_) δ 8.53–8.51 (m, 1H, pyr.), 8.45 (d, *J* = 2.4 Hz, 1H, pyr.), 7.92 (bs, 1H, NH), 7.37–7.28 (m, 2H, Ar), 7.07–6.97 (m, 2H, Ar), 4.60 (d, *J* = 6.5 Hz, 2H, CH_2_); ^13^C-NMR (75 MHz, CDCl_3_) δ 162.2 (d, *J* = 243.1 Hz), 161.9, 148.4, 145.9, 142.9, 140.6, 133.4 (d, *J* = 3.5 Hz), 129.6 (d, *J* = 9.2 Hz), 115.6 (d, *J* = 21.9 Hz), 43.0; Elemental analysis: calc. for C_12_H_9_ClFN_3_O (MW 265.67): 54.25% C, 3.41% H, 15.82% N; found 54.71% C, 3.76% H, 15.78% N.

*3-Chloro-N-(3-(trifluoromethyl)benzyl)pyrazine-2-carboxamide* (**12**). Pale yellow solid. Yield 55%; m.p. 79.2–81.5 °C; IR (ATR-Ge, cm^−1^): 3292, 1662 (C=O, CONH); ^1^H-NMR (500 MHz, DMSO) δ 9.44 (t, *J* = 6.0 Hz, 1H, NH), 8.72 (d, *J* = 2.4 Hz, 1H, pyr.), 8.65 (d, *J* = 2.4 Hz, 1H, pyr.), 7.74–7.72 (1H, m, Ar), 7.68–7.57 (m, 3H, Ar), 4.60 (d, *J* = 6.0 Hz, 2H, CH_2_); ^13^C-NMR (125 MHz, DMSO) δ 164.0, 147.9, 145.7, 145.3, 142.7, 140.4, 131.6, 129.6, 129.3 (q, *J* = 31.4 Hz), 124.4 (q, *J* = 272.5 Hz), 123.9 (q, *J* = 3.8 Hz), 42.0; Elemental analysis: calc. for C_13_H_9_ClF_3_N_3_O (MW 315.68): 49.46% C, 2.87% H, 13.31% N; found 49.12% C, 3.17% H, 12.89% N.

*3-Chloro-N-(4-(trifluoromethyl)benzyl)pyrazine-2-carboxamide* (**13**). White solid. Yield 67%; m.p. 123.7–125.7 °C; IR (ATR-Ge, cm^−1^): 3299, 1660 (C=O, CONH); ^1^H-NMR (500 MHz, CDCl_3_) δ 8.54 (d, *J* = 2.4 Hz, 1H, pyr.), 8.46 (d, *J* = 2.4 Hz, 1H, pyr.), 8.05 (bs, 1H, NH), 7.62–7.57 (m, 2H, Ar), 7.50–7.45 (m, 2H, AA´, BB´, Ar), 4.70 (d, 2H, *J* = 6.4 Hz, CH_2_); ^13^C-NMR (125 MHz, CDCl_3_) δ 162.1, 148.5, 145.9, 142.7, 141.7, 140.6, 129.9 (q, *J* = 32.4 Hz), 128.0, 125.7 (q, *J* = 3.9 Hz), 124.0 (q, *J* = 271.9 Hz), 43.2; Elemental analysis: calc. for C_13_H_9_ClF_3_N_3_O (MW 315.68): 49.46% C, 2.87% H, 13.31% N; found 49.01% C, 3.34% H, 12.95% N.

*N-(2-Chlorobenzyl)-3-((2-chlorobenzyl)amino)pyrazine-2-carboxamide* (**5a**). White solid. Yield 39%; m.p. 90.8–91.9 °C; IR (ATR-Ge, cm^−1^): 3382, 3353, 1663 (C=O, CONH); ^1^H-NMR (500 MHz, CDCl_3_) δ 9.09 (bs, 1H, NH), 8.39 (t, *J* = 6.1 Hz, 1H, NH), 8.19 (d, *J* = 2.4 Hz, 1H, pyr.), 7.71 (d, *J* = 2.4 Hz, 1H, pyr.), 7.45–7.36 (m, 4H, Ar), 7.28–7.23 (m, 2H, Ar), 7.23–7.18 (m, 2H, Ar), 4.82 (d, *J* = 6.1 Hz, 2H, CH_2_), 4.72 (d, *J* = 6.3 Hz, 2H, CH_2_); ^13^C-NMR (126 MHz, CDCl_3_) δ 166.40, 154.52, 146.50, 136.27, 135.40, 133.64, 133.57, 129.96, 129.70, 129.58, 129.45, 129.17, 128.90, 128.35, 127.06, 126.73, 126.46, 42.12, 41.04; Elemental analysis: calc. for C_19_H_16_Cl_2_N_4_O (MW 387.26): 58.93% C, 4.16% H, 14.47% N; found 58.69% C, 4.07% H, 14.21% N.

*N-(3-Chlorobenzyl)-3-((3-chlorobenzyl)amino)pyrazine-2-carboxamide* (**6a**). Yellow solid. Yield 62%; m.p. 68.7–70.4 °C; IR (ATR-Ge, cm^−1^): 3377, 3337, 1652 (C=O, CONH); ^1^H-NMR (500 MHz, CDCl_3_) δ 9.04 (t, *J* = 6.3 Hz, 1H, NH), 8.32 (bs, 1H, NH), 8.20 (d, *J* = 2.4 Hz, 1H, pyr.), 7.71 (d, *J* = 2.4 Hz, 1H, pyr.), 7.37–7.33 (m, 2H, Ar), 7.31–7.21 (m, 6H, Ar), 4.71 (d, *J* = 6.0 Hz, 2H, CH_2_), 4.58 (d, *J* = 6.3 Hz, 2H, CH_2_); ^13^C-NMR (126 MHz, CDCl_3_) δ 166.45, 154.46, 146.66, 141.14, 140.07, 134.57, 134.34, 130.09, 129.98, 129.76, 127.69, 127.65, 127.56, 127.24, 126.25, 125.70, 125.53, 43.73, 42.55; Elemental analysis: calc. for C_19_H_16_Cl_2_N_4_O (MW 387.26): 58.93% C, 4.16% H, 14.47% N; found 59.16% C, 4.17% H, 14.20% N.

*N-(4-Chlorobenzyl)-3-((4-chlorobenzyl)amino)pyrazine-2-carboxamide* (**7a**). Pale yellow solid. Yield 77%; m.p. 150.1–151.4 °C; IR (ATR-Ge, cm^−1^): 3375, 3346, 1651 (C=O, CONH); ^1^H-NMR (300 MHz, CDCl_3_) δ 8.94 (bs, 1H, NH), 8.20 (bs, 1H, NH), 8.10 (s, 1H, pyr.), 7.60 (s, 1H, pyr.), 7.32–7.12 (m, 8H, Ar), 4.59 (d, *J* = 5.9 Hz, 2H, CH_2_), 4.47 (d, *J* = 5.9 Hz, 2H, CH_2_); ^13^C-NMR (75 MHz, CDCl_3_) δ 166.4, 154.5, 146.6, 137.5, 136.5, 133.3, 132.8, 130.0, 129.0, 128.8, 128.6, 126.3, 43.6, 42.4; Elemental analysis: calc. for C_19_H_16_Cl_2_N_4_O (MW 387.26): 58.93% C, 4.16% H, 14.47% N; found 58.68% C, 4.24% H, 14.16% N.

*N-(3,4-Dichlorobenzyl)-3-((3,4-dichlorobenzyl)amino)pyrazine-2-carboxamide* (**9a**). Yellow solid. Yield 67%; m.p. 101.9–104.7 °C; IR (ATR-Ge, cm^−1^): 3368, 3317, 1666 (C=O, CONH); ^1^H-NMR (500 MHz, CDCl_3_) δ 9.03 (bs, 1H, NH), 8.34 (bs, 1H, NH), 8.20 (d, *J* = 2.4 Hz, 1H, pyr.), 7.74 (d, *J* = 2.5 Hz, 1H, pyr.), 7.46–7.37 (m, 4H, Ar), 7.28–7.18 (m, 2H, Ar), 4.68 (d, *J* = 6.0 Hz, 2H, CH_2_), 4.56 (d, *J* = 6.3 Hz, 2H, CH_2_); ^13^C-NMR (126 MHz, CDCl_3_) δ 166.46, 154.29, 146.61, 139.40, 138.30, 132.78, 132.49, 131.59, 131.00, 130.66, 130.42, 130.34, 129.48, 129.38, 126.91, 126.77, 126.22, 43.24, 42.06; Elemental analysis: calc. for C_19_H_14_ Cl_4_N_4_O (MW 456.15): 50.03% C, 3.09% H, 12.28% N; found 50.09% C, 3.22% H, 11.84% N.

*N-(2-Fluorobenzyl)-3-((2-fluorobenzyl)amino)pyrazine-2-carboxamide* (**10a**). Pale yellow solid. Yield 84%; m.p. 94.8–95.9 °C; IR (ATR-Ge, cm^−1^): 3375, 3349, 1652 (C=O, CONH); ^1^H-NMR (500 MHz, CDCl_3_) δ 9.00 (t, *J* = 5.5 Hz, 1H, NH), 8.30 (t, *J* = 6.1 Hz, 1H, NH), 8.19 (d, *J* = 2.2 Hz, 1H, pyr.), 7.69 (d, *J* = 2.2 Hz, 1H, pyr.), 7.42–7.36 (m, 2H, Ar), 7.31–7.21 (m, 2H, Ar), 7.15–7.08 (m, 4H, Ar), 4.78 (d, *J* = 5.5 Hz, 2H, CH_2_), 4.66 (d, *J* = 6.1 Hz, 2H, CH_2_); ^13^C-NMR (125 MHz, CDCl_3_) δ 166.4, 161.0 (d, *J* = 247.0 Hz), 154.5, 146.5, 130.0 (d, *J* = 3.9 Hz), 129.9, 129.5 (d, *J* = 3.9 Hz), 129.3 (d, *J* = 7.7 Hz), 128.7 (d, *J* = 7.7 Hz), 126.4, 125.9 (d, *J* = 14.3 Hz), 125.0 (d, *J* = 14.3 Hz), 124.3 (d, *J* = 3.8 Hz), 124.0 (d, *J* = 2.9 Hz), 115.5 (d, *J* = 19.1 Hz), 115.3 (d, *J* = 19.1 Hz), 38.1 (d, *J* = 4.8 Hz), 37.1 (d, *J* = 3.9 Hz); Elemental analysis: calc. for C_19_H_16_F_2_N_4_O (MW 354.36): 64.40% C, 4.55% H, 15.81% N; found 64.24% C, 4.49% H, 15.97% N.

*N-(4-Fluorobenzyl)-3-((4-fluorobenzyl)amino)pyrazine-2-carboxamide* (**11a**). Yellow-brown solid. Yield 75%; m.p. 97.1–99.1 °C; IR (ATR-Ge, cm^−1^): 3378, 3345, 1650 (C=O, CONH); ^1^H-NMR (500 MHz, CDCl_3_) δ 9.06–9.00 (m, 1H, NH), 8.31–8.25 (m, 1H, NH), 8.20 (d, *J* = 2.4 Hz, 1H, pyr.), 8.69 (d, *J* = 2.4 Hz, 1H, pyr.), 7.37–7.29 (m, 4H, Ar), 7.06–6.99 (m, 4H, Ar), 4.69 (d, *J* = 6.1 Hz, 2H, CH_2_), 4.56 (d, *J* = 6.1 Hz, 2H, CH_2_); ^13^C-NMR (125 MHz, CDCl_3_) δ 166.4, 162.2 (d, *J* = 246.1 Hz), 162.0 (d, *J* = 245.1 Hz), 154.5, 146.5, 134.6 (d, *J* = 3.9 Hz), 133.8 (d, *J* = 2.9 Hz), 129.7, 129.3 (d, *J* = 8.7 Hz), 129.1 (d, *J* = 8.5 Hz), 126.4, 115.6 (d, *J* = 22.0 Hz), 115.3 (d, *J* = 21.0 Hz), 43.6, 42.4; Elemental analysis: calc. for C_19_H_16_F_2_N_4_O (MW 354.36): 64.40% C, 4.55% H, 15.81% N; found 64.55% C, 4.41% H, 15.63% N.

*N-(4-(Trifluoromethyl)benzyl)-3-((4-(trifluoromethyl)benzyl)amino)pyrazine-2-carboxamide* (**13a**). Yellow solid. Yield 86%; m.p. 99.7–102.5 °C; IR (ATR-Ge, cm^−1^): 3388, 3334, 1652 (C=O, CONH); ^1^H-NMR (500 MHz, CDCl_3_) δ 9.08 (t, *J* = 5.6 Hz, 1H, NH), 8.38 (t, *J* = 6.3 Hz, 1H, NH), 8.20 (d, *J* = 2.4 Hz, 1H, pyr.), 7.73 (d, *J* = 2.4 Hz, 1H, pyr.), 7.64–7.57 (m, 4H, Ar), 7.50–7.45 (m, 4H, Ar), 4.79 (d, *J* = 5.6 Hz, 2H, CH_2_), 4.67 (d, *J* = 6.3 Hz, 2H, CH_2_); ^13^C-NMR (125 MHz, CDCl_3_) δ 166.6, 154.5 146.8, 143.2, 142.1, 130.3, 129.6 (q, *J* = 32.4 Hz), 129.6 (q, *J* = 32.4 Hz), 127.8, 127.6, 126.2, 125.7 (q, *J* = 3.8 Hz), 125.5 (q, *J* = 3.8 Hz), 121.1 (q, *J* = 276.9 Hz), 124.0 (q, *J* = 271.8 Hz), 43.9, 42.7; Elemental analysis: calc. for C_21_H_16_F_6_N_4_O (MW 454.38): 55.51% C, 3.55% H, 12.33% N; found 55.47% C, 3.68% H, 12.17% N.

### 3.4. Methods

#### 3.4.1. Determination of Lipophilicity by HPLC (log *k*)

Log *k* was determined using an Agilent Technologies 1200 SL liquid chromatograph with Diode-array Detector SL G1315C (Agilent Technologies Inc., Colorado Springs, CO, USA), with pre-column ZORBAX XDB-C18 5 μm, 4 mm × 4 mm, Part No. 7995118-504 (Agilent Technologies Inc.) and column ZORBAX Eclipse XDB-C18 5 μm, 4.6 mm × 250 mm, Part No. 7995118-585 (Agilent Technologies Inc.). The separation process was controlled by Agilent ChemStation, version B.04.02, extended by a spectral module (Agilent Technologies Inc.). The mobile phase consisted of MeOH (HPLC grade, 70%) and H_2_O (HPLC-Milli-Q Grade, 30%).

The flow rate was 1.0 mL/min, samples were injected in a volume of 20 μL, and the column temperature was 30 °C. A value of 210 nm as the detection wavelength and 270 nm as the monitor wavelength were used. Retention times (t_R_) were measured in minutes. The dead time of the system (t_D_) was determined as the retention time of the KI methanol solution. Capacity factors k for individual compounds were calculated according to the formula *k* = (t_R_ − t_D_)/t_D_. Log *k*, calculated from the capacity factor k, as used as the lipophilicity index converted to log scale.

#### 3.4.2. Evaluation of In Vitro Antimycobacterial Activity

Microdilution panel method. Tested strains *Mycobacterium tuberculosis* H37Rv CNCTC My 331/88, *M. kansasii* Hauduroy CNCTC My 235/80, and *M. avium* ssp. *avium* Chester CNCTC My 80/72 were obtained from the Czech National Collection of Type Cultures (CNCTC), National Institute of Public Health, Prague, Czech Republic. Middlebrook 7H9 broth (Sigma-Aldrich, Steinheim, Germany) enriched with the 0.4% of glycerol (Sigma-Aldrich) and 10% of OADC supplement (oleic acid, albumin, dextrose, catalase; Himedia, Mumbai, India) of declared pH = 6.6 was used for cultivation. Tested compounds were dissolved and diluted in DMSO and mixed with broth (25 μL of DMSO solution in 4.475 mL of broth) and placed (100 μL) into microplate wells. Mycobacterial inocula were suspended in isotonic saline solution and the density was adjusted to 0.5–1.0 McFarland. These suspensions were diluted by 10^−1^ and used to inoculate the testing wells, adding 100 μL of suspension to 100 μL of the DMSO/broth solution of tested compound. Final concentrations of the tested compounds in wells were 100, 50, 25, 12.5, 6.25, 3.13, and 1.56 μg·mL^−1^. Isoniazid was used as the positive control (inhibition of growth). The negative control consisted of broth plus DMSO. A total of 30 μL of Alamar Blue working solution (1:1 mixture of 0.1% resazurin sodium salt (aq. sol.) and 10% Tween 80) was added after five days of incubation. Results were then determined after 24 h of incubation. The minimum inhibitory concentration (MIC; μg·mL^−1^) was determined as the lowest concentration which prevented the blue to pink colour change. MIC values of isoniazid were 6.25–12.5 μg·mL^−1^ against *M. avium*, 3.13–12.5 μg·mL^−1^ against *M. kansasii*, and 0.2 μg·mL^−1^ against *M. tbc*.

#### 3.4.3. Antimycobacterial In Vitro Activity Screening Against *Mycobacterium Smegmatis*

An antimycobacterial assay was performed with fast growing *Mycobacterium smegmatis* CCM 4622 (ATCC 607) from the Czech Collection of Microorganisms (Brno, Czech Republic). The technique used for activity determination was a microdilution broth panel method using 96-well microtitration plates. The culturing medium was Middlebrook 7H9 (MB) broth (Sigma-Aldrich), enriched with 0.4% of glycerol (Sigma-Aldrich) and 10% of Middlebrook OADC growth supplement (Himedia, Mumbai, India). Tested compounds were dissolved in DMSO (Sigma-Aldrich), and the MB broth was then added to obtain a concentration of 2000 μg·mL^−1^. Standards used for activity determination were isoniazid (INH), rifampicin (RIF), and ciprofloxacin (CPX) (Sigma-Aldrich). Final concentrations were reached by binary dilution and the addition of mycobacterial suspension, and were set as 500, 250, 125, 62.5, 31.25, 15.625, 7.81, and 3.91 μg·mL^−1^, except for the standards of ciprofloxacin and rifampicin, where the final concentrations were 12.5, 6.25, 3.125, 1.56, 0.78, 0.39, 0.195, and 0.098 μg·mL^−1^. The final concentration of DMSO did not exceeded 2.5% (*v*/*v*) and did not affect the growth of *M. smegmatis*. Plates were also sealed with polyester adhesive film and incubated in the dark at 37 °C, without agitation. The addition of 0.01% solution of resazurin sodium salt followed after 48 h. This stain was prepared by dissolving resazurin sodium salt (Sigma-Aldrich) in deionised water, producing a 0.02% solution. Then, a 10% aqueous solution of Tween 80 (Sigma-Aldrich) was prepared. Both liquids were mixed up making use of the same volumes and filtered through a syringe membrane filter. Microtitration panels were then incubated for a further 4 h. Antimycobacterial activity was expressed as the minimal inhibition concentration (MIC) and the value was read on the basis of stain colour change (blue colour—active compound; pink colour—not active compound). The MIC values for the standards were in the range of 7.81–15.625 μg·mL^−1^ for INH, 0.78–1.56 μg·mL^−1^ for RIF, and 0.098–0.195 μg·mL^−1^ for CPX. All experiments were conducted in duplicate.

#### 3.4.4. Evaluation of In Vitro Antibacterial Activity

Microdilution broth method. Antibacterial evaluation was performed against eight bacterial strains from the Czech Collection of Microorganisms (Brno, Czech Republic) (*Staphylococcus aureus* CCM 4516/08, *Escherichia coli* CCM 4517, *Pseudomonas aeruginosa* CCM 1961) or clinical isolates from the Department of Clinical Microbiology, University Hospital and Faculty of Medicine in Hradec Králové, Charles University in Prague, Czech Republic (*Staphylococcus aureus* H 5996/08-methicilin resistant (MRSA), *Staphylococcus epidermidis* H 6966/08, *Enterococcus* sp. J 14365/08, *Klebsiella pneumoniae* D 11750/08, *Klebsiella pneumoniae* J 14368/08-ESBL positive). All strains were subcultured on Mueller-Hinton agar (MHA) (Difco/Becton Dickinson, Detroit, MI, USA) at 35 °C and maintained on the same medium at 4 °C. The compounds were dissolved in DMSO, and the antibacterial activity was determined in Mueller-Hinton liquid broth (Difco/Becton Dickinson), and buffered to pH 7.0. Controls consisted of medium and DMSO alone. The final concentration of DMSO in the test medium did not exceed 1% (*v*/*v*) of the total solution composition. The minimum inhibitory concentration (MIC), defined as the minimum concentration to prevent the visible growth compared to control, was determined after 24 and 48 h of static incubation at 35 °C. The standards were neomycin, bacitracin, penicillin G, ciprofloxacin, and phenoxymethylpenicillin.

#### 3.4.5. Evaluation of In Vitro Antifungal Activity

Antifungal evaluation was performed using a microdilution broth method against eight fungal strains (*Candida albicans* ATCC 44859, *C. tropicalis* 156, *C. krusei* E28, *C. glabrata* 20/I, *Trichosporon asahii* 1188, *Aspergillus fumigatus* 231, *Lichtheimia corymbifera* 272 and *Trichophyton mentagrophytes* 445). Compounds were dissolved in DMSO and diluted in a twofold manner with RPMI 1640 medium, with glutamine buffered to pH 7.0 (3-morpholinopropane-1-sulfonic acid). The final concentration of DMSO in the tested medium did not exceed 2.5% (*v*/*v*) of the total solution composition. Static incubation was performed in the dark and humid, at 35 °C, for 24 and 48 h (respectively 72 and 120 h for *Trichophyton mentagrophytes*). Drug-free controls were included. The standards were amphotericin B, voriconazole, nystatin, and fluconazole.

#### 3.4.6. Cytotoxicity Assays

The human liver hepatocellular carcinoma cell line HepG2 (passage 35–36), purchased from Health Protection Agency Culture Collections (ECACC, Salisbury, UK), was routinely cultured in Minimum Essential Eagle Medium MEM (Sigma-Aldrich) supplemented with 10% fetal bovine serum (PAA Laboratories GmbH, Pasching, Austria), 2mM L-glutamine solution (Sigma-Aldrich), and 1% non-essential amino acid solution (Sigma-Aldrich), in a humidified atmosphere containing 5% CO_2_ at 37 °C.

For subculturing, the cells were harvested after trypsin/EDTA (Sigma-Aldrich) treatment at 37 °C. To evaluate the cytotoxicity, the HepG2 cells treated with the tested substances were used as experimental groups, whereas untreated HepG2 cells served as control groups.

The HepG2 cells were seeded in a density of 1 × 10^4^ cells per well on a 96-well plate. The following day (24 h after seeding), they were treated with tested substances dissolved in DMSO (maximal incubation concentration of DMSO was 1%). The tested substances were prepared according to their solubility in DMSO, at incubation concentrations of 1–2500 μM. The treatment was carried out in a humidified atmosphere containing 5% CO_2_ at 37 °C, in triplicate, for 24 h. The controls representing 100% cell viability, 0% cell viability (the cells treated with 10% DMSO), no-cell controls, and vehiculum controls were incubated in triplicate, simultaneously. After 24 h exposure, the reagent from the kit CellTiter 96^®^ Aqueous One Solution Cell Proliferation Assay (Promega) was added, according to the recommendation of the manufacturer. After 2 h incubation at 37 °C in humidified, 5% CO_2_ atmosphere, the absorbance was recorded at 490 nm. Inhibitory curves were constructed for each compound, plotting incubation concentrations vs. percentage of absorbance relative to untreated control. The standard toxicological parameter IC_50_ was calculated by a nonlinear regression analysis of the inhibitory curves using GraphPad Prism software (version 6, GraphPad Software, Inc., La Jolla, CA, USA).

## 4. Conclusions

Thirteen new *N*-benzyl-3-chloropyrazine-2-carboxamide derivatives and ten *N*-benzyl-3-(benzylamino)pyrazine-2-carboxamide derivatives were prepared and analytically characterized. Further, antimycobacterial, antibacterial, and antifungal assays were performed. Compounds **1**–**13** presented in this paper were prepared for the purpose of completing series of 5- and 6-positional isomers, as reported previously. However, the chlorine shift to position 3 on the pyrazine ring led to the decrease of antimycobacterial activity compared to 5- or 6-positional isomers. Contrary to 5-Cl and 6-Cl positional isomers, *N*-benzyl-3-chloropyrazine-2-carboxamides exerted antibacterial activity against staphylococcal strains. The most effective compound was **5**, with micromolar activity against *Staphylococcus aureus* and *Staphylococcus epidermidis*. Interestingly, some initially unplanned side products with double benzyl substitution possessed better antimycobacterial activity than the intended *N*-benzyl-3-chloropyrazine-2-carboxamides. From compounds with double phenyl substitution, **1a** and **9a** exerted moderate antimycobacterial activity (tens of μM) and **3a** was moderately active against methicillin-resistant *Staphylococcus aureus* (tens of μM). Generally, the insertion of the second aromatic ring was beneficial to the activity against *M. tuberculosis* H37Rv, but contra-productive for activity against *M. smegmatis* and other tested bacteria. Importantly, the discussed active compounds were assayed for in vitro cytotoxicity with plausible results. 

## Figures and Tables

**Table 1:** Prepared structures, calculated (Clog*P*) and measured (log *k*) lipophilicity parameters, and antimycobacterial activity against *M. tuberculosis* H37Rv and *M. smegmatis*.

No.	R^1^	R^2^	MW	Clog*P*	Log *k*	MIC (μg·mL^−1^) (μM)	
						*M. tuberculosis*	*M. smegmatis*
**1**	2-CH_3_	-	261.71	2.004	0.009	>100	250
**1a**	2-CH_3_	2-CH_3_	346.43	4.862	1.459	12.5 (36)	> 500
**2**	4-CH_3_	-	261.71	2.054	0.033	>100	>500
**2a**	4-CH_3_	4-CH_3_	346.43	4.962	1.517	25 (72)	>250
**3**	4-OCH_3_	-	277.71	1.474	−0.202	25 (90)	125
**3a**	4-OCH_3_	4-OCH_3_	378.43	3.802	0.981	50 (132)	>500
**4**	2,4-diOCH_3_	-	307.73	1.563	−0.095	>100	>500
**5**	2-Cl	-	282.12	2.268	0.033	>100	250
**5a**	2-Cl	2-Cl	387.26	5.390	1.542	>100	>500
**6**	3-Cl	-	282.12	2.268	0.060	100	125
**6a**	3-Cl	3-Cl	387.26	5.390	1.515	>100	>500
**7**	4-Cl	-	282.12	2.268	0.082	>100	500
**7a**	4-Cl	4-Cl	387.26	5.390	1.533	>50	>250
**8**	2,4-diCl	-	316.57	2.981	0.375	>100	125
**9**	3,4-diCl	-	316.57	2.861	0.309	100	250
**9a**	3,4-diCl	3,4-diCl	456.15	6.576	1.997	12.5 (27)	>500
**10**	2-F	-	265.67	1.698	−0.154	>100	125
**10a**	2-F	2-F	354.36	4.250	1.108	>100	>500
**11**	4-F	-	265.67	1.698	−0.139	>100	250
**11a**	4-F	4-F	354.36	4.250	1.058	>100	>500
**12**	3-CF_3_	-	315.68	2.438	0.110	>100	125
**13**	4-CF_3_	-	315.68	2.438	0.145	>100	>500
**13a**	4-CF_3_	4-CF_3_	454.38	5.730	1.593	>100	>500
INH	-	-	-	-	-	0.2	7.8–15.6

**Table 2:** Notable antibacterial activities of the effective compounds and standards neomycin and phenoxymethylpenicillin (Penicillin V) against *Staphylococcus aureus*, methicillin-resistant *Staphyloc-occus aureus* (MRSA), and *Staphylococcus epidermidis*.

Compound	MIC (μM) after 24 h		
	*S. aureus*	MRSA	*S. epidermidis*
**2**	125	>500	500
**3**	31.25	125	250
**3a**	15.62	62.5	>500
**4**	125	>500	>500
**5**	7.81	500	15.62
**8**	125	250	125
**9**	62.5	250	31.25
**13**	31.25	500	62.5
**13a**	62.5	>500	>500
Penicillin V	0.12	62.5	3.9
Neomycin	1.95	0.98	7.81

**Table 3:** Cytotoxicity of tested substances in HepG2 cells.

Compound	IC_50_ (μM)
**5**	847.2
**1a**	>250 *
**3a**	>100 *
**9a**	>100 *

* Measurement at higher concentration not reproducible due to the precipitation of the tested compound in the cell culture medium.

## Supplementary Materials

The docking studies are available online.
