# Supplementary material for: 3-Substituted N-Benzylpyrazine-2-carboxamide Derivatives: Synthesis, Antimycobacterial and Antibacterial Evaluation
Source: Molecules. 2017 Mar 21;22(3):495. doi: 10.3390/molecules22030495 (PMC6155222; doi:10.3390/molecules22030495)
Supplement: Supplementary file 1 [file molecules-22-00495-s001.pdf]

3-Substituted *N*-Benzylpyrazine-2-Carboxamide Derivatives: Synthesis, Antimycobacterial and Antibacterial Evaluation

Lucia Semelková [1,*], Ondřej Janďourek [1], Klára Konečná [1], Pavla Paterová [2], Lucie Navrátilová [1], František Trejtnar [1], Vladimír Kubíček [1], Jiří Kuneš [1], Martin Doležal [1] and Jan Zitko [1,*]

[1] Charles University, Faculty of Pharmacy in Hradec Králové, Heyrovského 1203, 500 05 Hradec Králové, Czech Republic; E-Mails: JANDO6AA@faf.cuni.cz (O.J.) konecna@faf.cuni.cz (K.K.); navratl2@faf.cuni.cz (L.N.); trejtnarf@faf.cuni.cz (F.T.); kubicek@faf.cuni.cz (V.K.); kunes@faf.cuni.cz (J.K.); dolezalm@faf.cuni.cz (M.D.)

[2] Department of Clinical Microbiology, University Hospital, Sokolská 581, 500 05 Hradec Králové, Czech Republic; pavla.paterova@fnhk.cz (P.P.)

* Correspondence: semelkol@faf.cuni.cz (L.S.); jan.zitko@faf.cuni.cz (J.Z.); Tel.: +420-495-067-275 (L.S.)

Supplementary Material

Docking Studies

1. Results and Discussion

Most of the compounds presented in this paper with *in vitro* activity against *M. tuberculosis* were disubstituted derivatives with two large benzyl substituents on two adjacent positions of the pyrazine core. We were interested to find out whether such sterically demanding derivatives would be able to fit in the active site of InhA in a manner similar to smaller PZA derivatives with single aryl substituent. Therefore, we performed molecular docking of the most active dibenzyl derivative **9a** into various conformations of InhA, differing in the size of the active site cavity, which is formed by the highly flexible substrate-binding loop (Fig. S1). Not surprisingly, **9a** was not able to fit into closed conformations of InhA (pdb: 2X23; 3FNF) and did not show the expected ligand-receptor interactions. On the other hand, when opened conformation of InhA receptor was used (pdb: 4R9S, 4TZK, or 5G0S), we were able to identify two different binding modes for **9a** with scores similar to the score of the co-crystalized ligands and, more importantly, with ligand-receptor interactions known to be typical for InhA inhibitors. See Table S1.

The **first binding mode** (Fig. S2) was similar to the one suggested for simpler *N*-benzylaminopyrazine-2-carboxamides, where the *H*-bond accepting moiety was the carbonyl oxygen of the carboxamide moiety. In our case, the best pose of **9a** was further stabilized by *H*-bond interaction of carboxamide hydrogen (donor) to sulphur of Met199 (acceptor). The docking score for **9a** was -9.0 kcal/mol compared to redocked ligand with -10.72 kcal/mol. However, when we use the docking score weighted by the number of heavy atoms of the ligand ('ligand efficiency'), ligand **9a** can be considered more efficient fragment than the original co-crystalized ligand (see Table S1).

In the **second binding mode** (Fig. S3), the *H*-bond accepting role of **9a** was played by the N-1 nitrogen of the pyrazine core. Consequently, the carbonyl oxygen remained available for the intramolecular *H*-bond with NH of the benzylamino substituent. This intramolecular *H*-bond was present in all low energy conformations of **9a** as determined by low mode molecular dynamics conformational search. Therefore, the availability of carbonyl oxygen for intramolecular *H*-bond increases the stability of this binding mode. However, the N-1 nitrogen of the pyrazine core can accept only one *H*-bond. Interestingly, the interaction between ligand and phenolic hydroxyl of Tyr158 has the form of arene-H interaction (see Fig. S3).

In both binding modes, the ligand **9a** fits tightly to hydrophobic areas of the binding pocket as visible from the contour lines on the ligand-interaction diagrams (Fig. S4).

2. Conclusions

Although the results of molecular docking are not enough to confirm the inhibition of InhA as the mechanism of action of derivative **9a**, we have shown that even such sterically demanding derivatives are able to mimic poses and interactions of known InhA inhibitors. Selection of suitable structure of InhA with open conformation was crucial in this docking study.

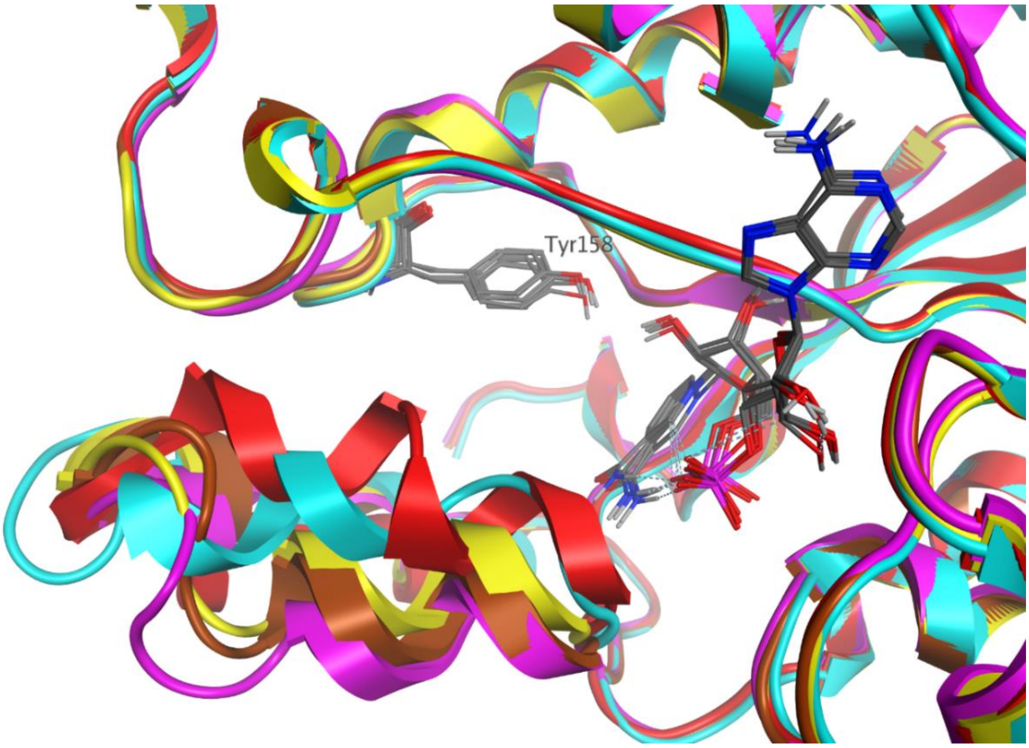

Fig. S1. Superposition of various conformations of mycobacterial enoyl-ACP-reductase (InhA). PDB codes: magenta - 4R9S, brown - 4TZK, yellow - 5G0S, cyan - 3FNF, red - 2X23.

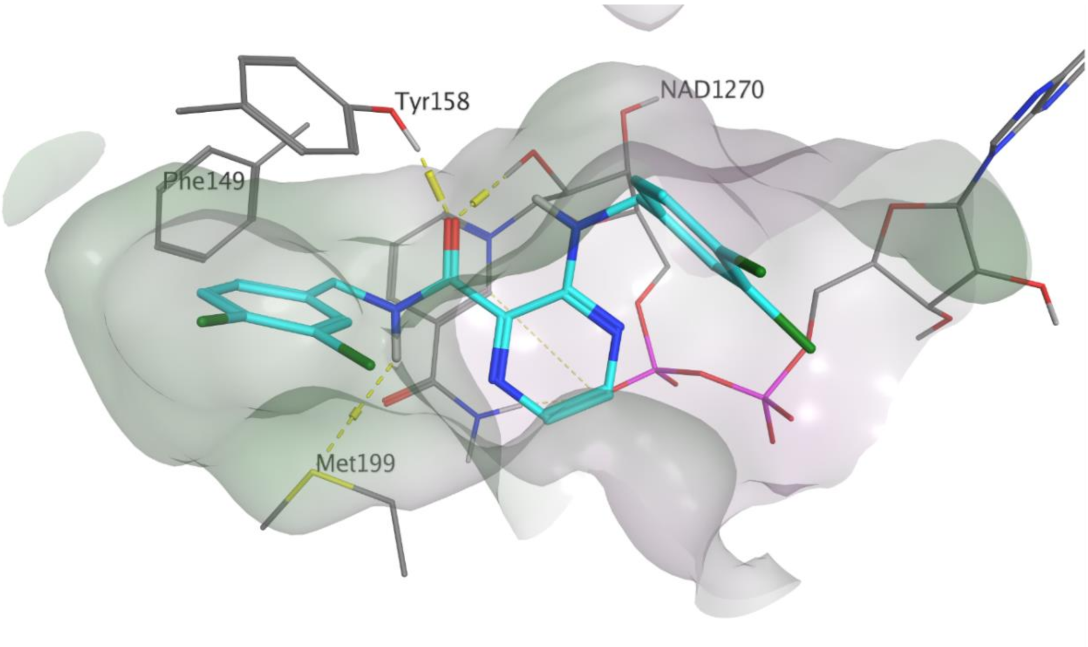

Fig. S2. Predicted binding mode of **9a** in mycobacterial enoyl-ACP-reductase (InhA; pdb: 5G0S). Binding mode 1.

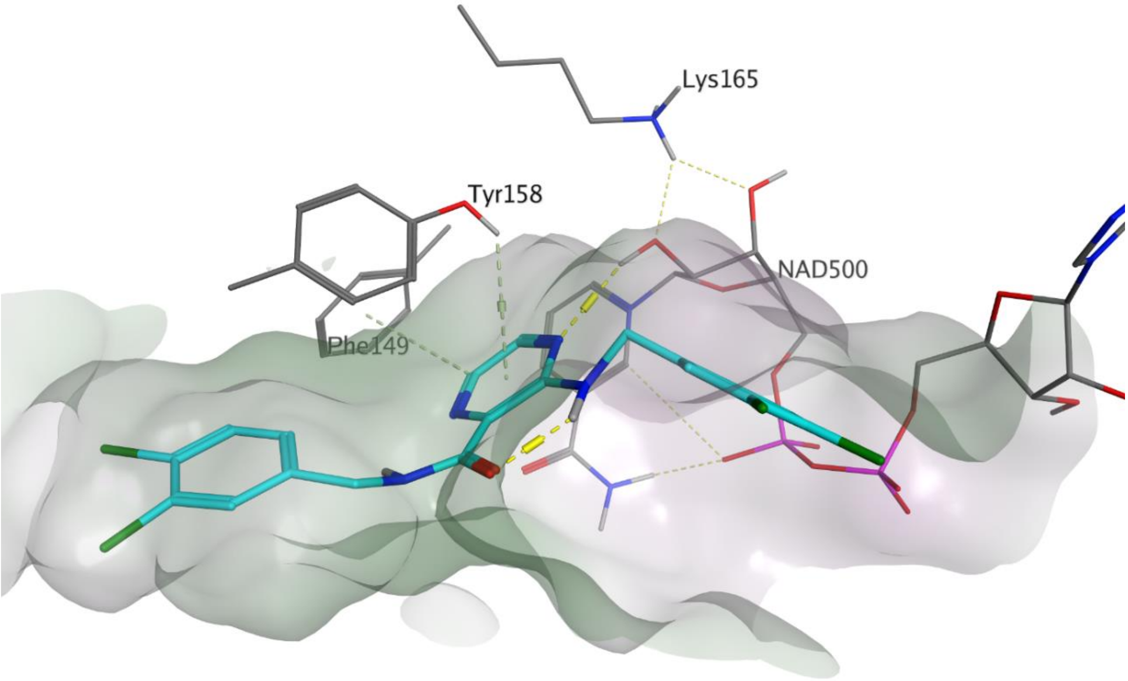

Fig. S3. Predicted binding mode of **9a** in mycobacterial enoyl-ACP-reductase (InhA; pdb: 4TZK). Binding mode 2.

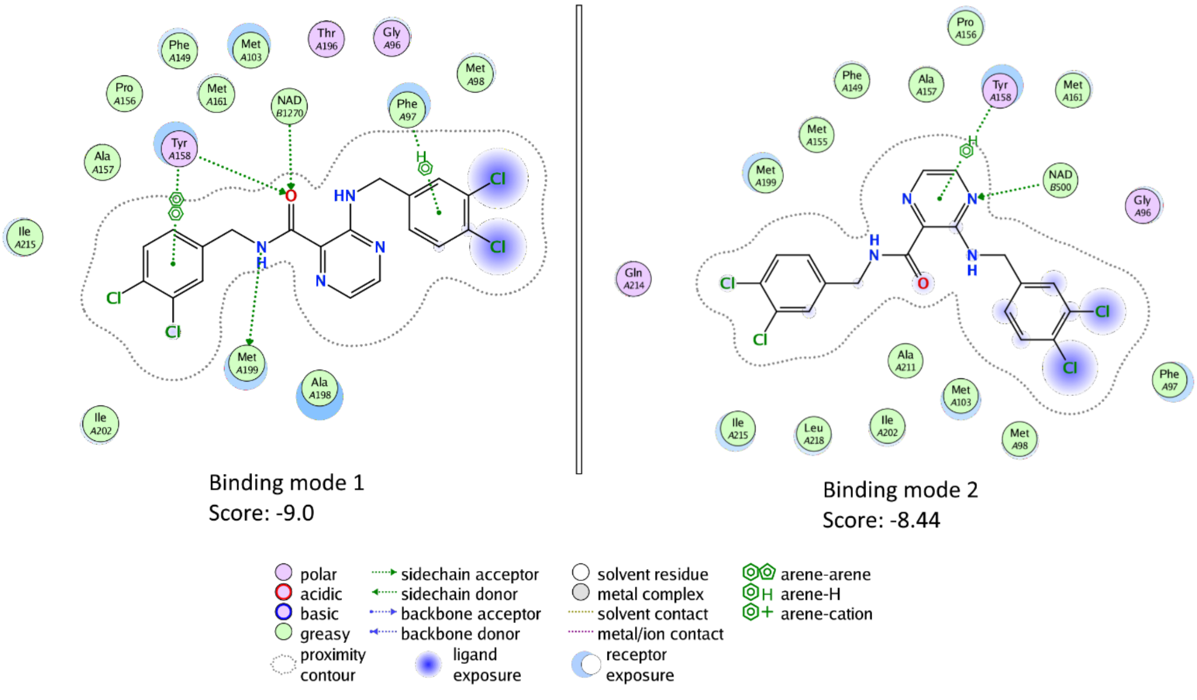

Fig. S4. Ligand interaction diagrams for **9a**. Binding mode 1 – pdb: 5G0S; binding mode 2 – pdb: 4TZK

Table S1. Docking scores, 'ligand efficiencies' and interactions of **9a** in various forms on InhA in comparison with redocked original ligands

Receptor	Ligand 9a			Redocked ligand			
PDB ID	Best score (kcal/mol)	Interactions to Tyr158 and/or 2′-OH	LE	Score (kcal/mol)	RMSD	No. of heavy atoms	LE
2X23	-7.82	no	-0.279	-10.02	0.10	21	-0.477
3FNF	-8.45	no	-0.302	-8.93	0.27	23	-0.388
5G0S	-9.00	yes, binding mode 1	-0.321	-10.72	0.97	42	-0.255
4TZK	-8.44	yes, binding mode 2	-0.301	-9.17	0.31	23	-0.399
4R9S	-8.30	yes, binding mode 1	-0.296	-8.42	0.08	23	-0.366

LE – 'ligand efficiency', calculated as the docking score divided by the number of heavy atoms (atoms other than hydrogen) of the ligand. **9a** has 28 heavy atoms.

Note

The primary objective of this molecular docking study was to determine whether sterically demanding derivatives with two large benzyl substituents at the adjacent positions of the pyrazine core would be theoretically compatible with the active site cavity of mycobacterial enoyl-ACP-reductase. This was confirmed as documented above. However, as suggested by a reviewer of the manuscript, we probed the differences in binding between corresponding 3-chloro derivatives (one benzyl substituent) and 3-benzylamino derivatives (two benzyl substituents). Using the same methodology as described below, we performed docking of the most active 3-chloro derivative **3** along 3-benzylamino derivatives (**1a**, **2a**, **3a**, **9a**) into various forms of InhA. The results can be summarized as follows:

- The smaller molecule of **3** had more binding modes then sterically demanding **3a**, especially in open InhA conformations.
- The docking score (and ranking) of **3** was worse than the score of **3a**, as well as other dibenzyl derivatives (**1a**, **2a**, **9a**).
- With one exception, no predicted poses of **3** formed critical *H*-bond interactions to neither Tyr158 nor 2′-OH of the ribose of NAD^+.
- In the best scored pose of **3** in InhA pdb: 2X23, the oxygen of the carboxamide moiety accepted the hydrogen of 2′-OH of the ribose of NAD^+. However, in this pose the plane of the carboxamide moiety was significantly rotated out of the plane of the pyrazine core. Many scoring functions would penalize this non-planarity of the conjugated carboxamide moiety.

Judging only from the docking score and ligand-receptor interaction patterns, 3-benzylamino derivatives should be better inhibitors of InhA than 3-chloro derivatives. However, this molecular docking study alone is not enough to suggest the inhibition of InhA as a mechanism of action of neither of the structural classes.

3. Experimental

3.1. Molecular docking to mycobacterial enoyl-ACP-reductase

All *in silico* calculations and production of figures were performed in Molecular Operating Environment (MOE), 2016.08 (Chemical Computing Group Inc., Montreal, QC, Canada) using the Amber10:EHT forcefield. Coordinates of various confirmations of *M. tuberculosis* enoyl-ACP-reductase (InhA) were downloaded from the PDB database (pdb codes sorted from the most opened to most closed no conformations were 4R9S, 4TZK, 5G0S, 3FNF, 2X23. The complexes were superpositioned in space based on the protein sequence alignment (see Fig. S1). Receptors were prepared by MOE QuickPrep functionality with default settings. This included correction of structural errors, addition of hydrogens, calculation of partial charges, 3D optimisation of *H*-bond network (Protonate3D), deletion of water molecules further then 4.5 Å from any receptor or ligand atom, and restrained minimization of ligand and pocket residues within 8 Å from the ligand. We identified no waters important in mediating ligand-receptor or ligand-cofactor interactions), so all water molecules were removed. NAD^+ cofactor was defined as a part of the receptor.

Ligand **9a** was created by MOE Builder. Partial charges were computed and the ligand was minimized by conjugate gradient method to RMS gradient of 0.1 kcal.mol^{-1} Å^{-1}. The minimized structure served as an input for conformational search by low mode molecular dynamics. The search generated 61 non-redundant conformations within 7 kcal/mol energy window. This ensemble was then used as the input for docking.

The docking was focused on the pocket defined as residues within 5 Å from the co-crystalized ligand. Details of the MOE docking protocol setup: **Docking stage** - Placement method: Triangle Matcher; Score: London dG; retain 30 poses. **Refinement stage** – Rigid receptor; Score: GBVIWSA dG; retain 5 poses. The suitability of the docking protocol was confirmed by redocking of the randomized conformations of co-crystalized ligands (see Table S1 for RMSD between redocked and original poses).
